# Real-Time Reporting of Small Operational Failures in Nursing Care

**DOI:** 10.1155/2016/8416158

**Published:** 2016-11-08

**Authors:** Kathleen R. Stevens, Robert L. Ferrer

**Affiliations:** ^1^School of Nursing, University of Texas Health Science Center at San Antonio, San Antonio, TX, USA; ^2^Department of Family and Community Medicine, University of Texas Health Science Center at San Antonio, San Antonio, TX, USA

## Abstract

Addressing microsystem problems from the frontline holds promise for quality enhancement. Frontline providers are urged to apply quality improvement; yet no systematic approach to problem detection has been tested. This study investigated a self-report approach to detecting operational failures encountered during patient care.* Methods*. Data were collected from 5 medical-surgical units over 4 weeks. Unit staff documented operational failures on a small distinctive Pocket Card. Frequency distributions for the operational failures in each category were calculated for each hospital overall and disaggregated by shift. Rate of operational failures on each unit was also calculated.* Results*. A total of 160 nurses participated in this study reporting a total of 2,391 operational failures over 429 shifts. Mean number of problems per shift varied from 4.0 to 8.5 problems with equipment/supply problems being the most commonly reported category.* Conclusions*. Operational failures are common on medical-surgical clinical units. It is feasible for unit staff to record these failures in real time. Many types of failures were recognized by frontline staff. This study provides preliminary evidence that the Pocket Card is a feasible approach to detecting operational failures in real time. Continued research on methodologies to investigate the impact of operational failures is warranted.

## 1. Introduction

Nurses are the largest sector of the healthcare workforce and, as such, constitute most of what human factors experts call the “sharp end” of the healthcare system, the segment in direct contact with patients. This is especially true in hospitals, the sharpest, most hazardous, site of patient care [1], where 63.2% of nurses (2.8 million RNs) were employed in 2010 [2]. It is long-recognized that nurses are thus well-positioned to be leaders in the ongoing transformation of American healthcare triggered by the release in 2000 of the Institute of Medicine's To Err Is Human report [3] and emphasized in subsequent reports, Keeping Patients Safe: Transforming the Work Environment of Nurses [4] and Future of Nursing: Leading Change, Advancing Health (2011) [5].

Understanding nursing units from a complex adaptive systems (CAS) perspective, with its emphasis on patterns of relationships, has the potential to transform the approach to hospital quality improvement from a mechanistic enterprise to one that respects the diversity, talents, and connectedness of the whole healthcare team. The CAS framework helps to explain the connection between small operational failures that occur during the delivery of care and the negative or positive impact these have on quality and safety. Most adverse events in healthcare originate from small process failures that are common enough to be taken for granted [6, 7]. Although these process failures include both errors and “problems,” task interruptions due to something or someone not being available when needed, problems are far more common yet have drawn far less attention [7, 8]. In fact, problems occur about once per hour per nurse on hospital units, and 95% of problems are managed through workarounds (alternate ways to achieve a goal) rather than system corrections [8, 9].

How problems are managed, therefore, is considered to be an important determinant of a hospital's organizational culture for quality of care [9, 10]. Moreover, failure to use the frontline workers' knowledge of quality problems discards an important source of organizational intelligence for quality improvement [11].

Operational failures in nursing practice can be defined as task interruptions due to something or someone not being available when needed. More formally, Tucker has defined theses as “inability of the work system to reliably provide information, services, and supplies when, where, and to whom needed.” [12]. Operational failures are met with workarounds and are distinct from errors or mistakes [7] because the resulting workaround is intentional, an effort to produce the desired end result in spite of an obstacle. The study aim was to investigate the feasibility and effectiveness of an approach to engaging frontline clinicians in the detection of operational failures during daily patient care and to describe the failures they detected. Here, we report on the development and pilot testing of a data collection approach for frontline nursing staff to report on small operational failures in real time during daily patient care. We also briefly describe how nursing units and managers responded to the operational failure data. This was the initial phase of a study designed to test the hypothesis that frontline nursing staff's reporting of operational failures, combined with a systematic response at the clinical unit and hospital level, could lead to improvements in the quality of hospital care.

## 2. Methods

This was a descriptive cross-sectional study of the operational failures reported by frontline nursing staff during their work shifts on medical-surgical clinical units at two large South Texas hospitals anonymously referred in this report as Hospitals A and B. Three medical-surgical clinical units at Hospital A (A1, A2, and A3) and two medical-surgical units at Hospital B (B1 and B2) were selected for participation. Clinical units were purposively sampled to represent neither the best nor the worst performing units at their institutions as defined by nursing-sensitive outcomes. After the clinical units were identified, study staff discussed the research protocol with unit managers and nursing staff and obtained individual informed consent from participating staff.

**Figure 1:**
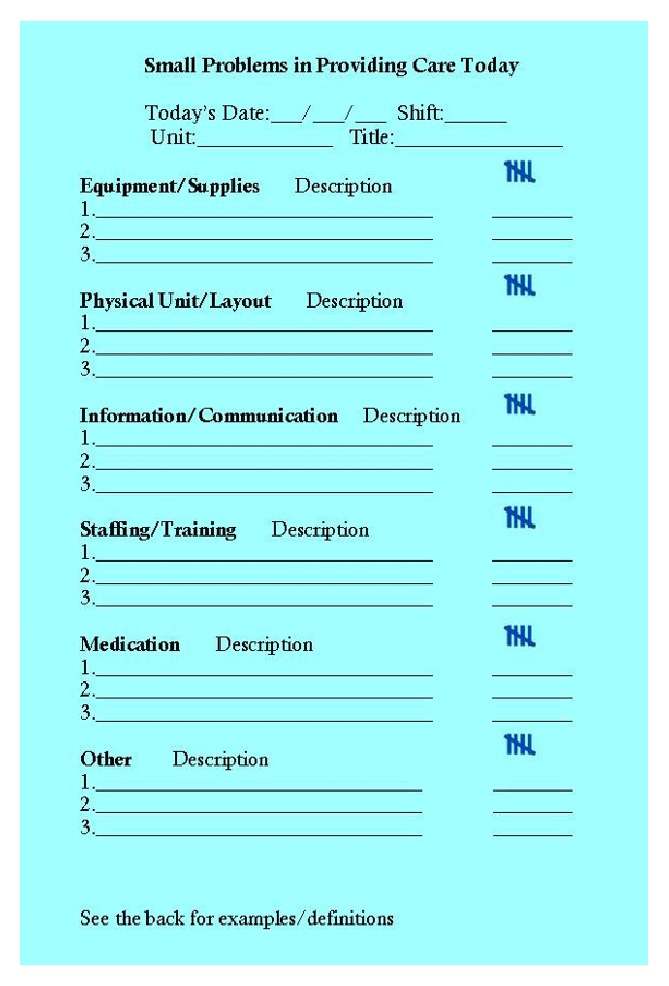
STAR Pocket Card (2008 copyright Stevens & Ferrer).

A low-tech data collection instrument was designed for detecting operational failures in real time. The instrument was designed as a structured, partially open response checklist (Figure 1) on a “Pocket Card.” To achieve content validity, checklist items were derived from direct observations of operational failures previously reported by Tucker and Spear (2006) [12]. Added to this content validity, face validity of this instrument was established through interviews with participating nursing staff. Following this instrument development, six general categories of operational failures were listed on the cards: equipment, physical unit, information or communication, staffing, medications, and “other.”

Nursing staff were introduced to the concept of operational failures and workarounds through staff meetings held at each clinical unit. Operational failure examples were also provided. Examples of descriptions in each category include the following: equipment, “no available linens” and “IVAC not charged”; physical unit, “not enough room to fit needed equipment around patient”; information/communication, “order not reported by previous shift”; staffing “no available help for patient lifting”; medications, “medications missing from automated medication dispenser”; other, “food trays not removed.” Nursing staff were instructed to record operational failures as they occurred, in real time, with a short description of the failure in the appropriate category and a hash mark to indicate the frequency of that failure. The shift was identified by the date, shift time, clinical unit, and title (RN, LVN, PCA, etc.) of the respondent.

During the data collection period, nursing staff were prompted to complete and submit cards by nurse mangers, study staff, and flyers posted in common areas in the clinical unit. At the end of each work shift, each nursing staff member deposited his/her Pocket Cards in locked boxes on their respective clinical unit. No personal identifiable information about the nursing staff was recorded.

Pocket Cards were then collected by the study team for entry into an electronic database. Using the list of small problems described above, entries on individual cards were assigned codes and entered along with notations for the unit, date, and shift that small problem reports could be aggregated to the desired level for reporting. Tables and charts displaying the frequencies of operational failures were distributed to the nursing units and senior nurse managers.

After data collection was completed, a facilitator from the study team worked with nursing units to organize a response to the Card data. The facilitator encouraged units to form improvement teams and presented their unit's data on frequencies of operational failures. Units then discussed and planned possible improvement projects, supported by the facilitator, who offered units a toolbox of quality improvement methods for potential use.

The study was approved by the Institutional Review Board of the University of Texas Health Science Center at San Antonio as well as the IRBs of the participating hospitals.

## 3. Analysis

After the Pocket Cards were collected from the clinical units, data were entered into an electronic database for analysis. Because each of the six categories contained an array of reported operational failures, the data were further classified into subcategories. Frequency distributions for the operational failures in each category and subcategory were tabulated for each hospital overall and then disaggregated by unit and shift. Rate of operational failures on each unit was also calculated by dividing the total number of reported problems by the number of work shift hours recorded on the cards. Box plots were used to examine the distribution of the data between day and night shifts. Data are reported as mean ± standard deviation.

## 4. Results

### 4.1. Description of Participating Hospitals

Hospital A was a for-profit, non-Magnet agency licensed for 600+ beds. Hospital B was a 400+ beds, not-for-profit agency recognized by the American Nurses Credentialing Center as a Magnet facility [13] and had implemented the Transforming Care at the Bedside (TCAB) program [14–16].

### 4.2. Participants

The 5 participating medical-surgical units ranged in size from 42 to 95 nursing staff. In all, of the 279 staff members, 160 consented nursing staff (57.3%) returned Pocket Cards for 429 shifts, representing a 57.3% response rate. Of the participating staff, 66.4% were registered nurses (RN), 4.3% were licensed vocational nurses (LVN), and 16% were patient care assistants or unit clerks. The study participation rates across the 5 units are presented in Table 1.

### 4.3. Frontline Detection of Operational Failures

A total of 2,391 operational failures were reported over 429 shifts. The frequency of failures reported over a single shift ranged from 0 to 40 per nurse. The distribution was positively skewed with an overall mean of 5.6 (95% CI: 4.9–6.2) operational failures reported per 12-hour shift and a median of 3. The mean number of problems varied from 4.0 to 8.5 problems per 12-hour shift. An ANOVA predicting problems/12 hours by unit rejects the null hypothesis for equal means (Table 2).

**Figure 2:**
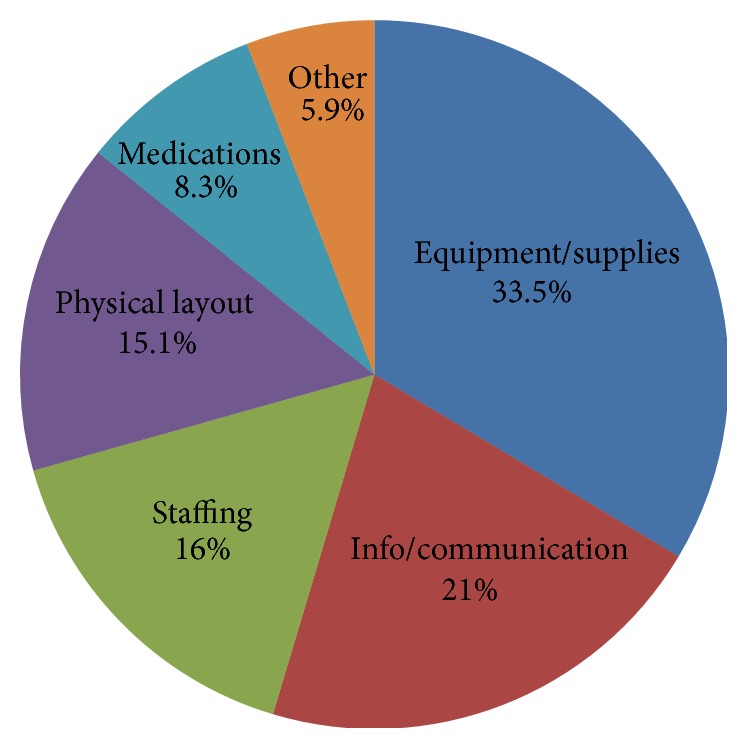
Distribution of operational failures by category (*n* = 2,391).

Distribution of operational failures across the 6 categories is presented in Figure 2. Briefly, of the 2,391 total operational failures reported, equipment/supply problems (*n* = 803; 33.5%) were the most frequently reported, followed by information/communication (*n* = 503; 21%), staffing (*n* = 383; 16%), physical unit (*n* = 362; 15.1%), medication (*n* = 200; 8.3%), and other (*n* = 140; 5.9%).

**Figure 3:**
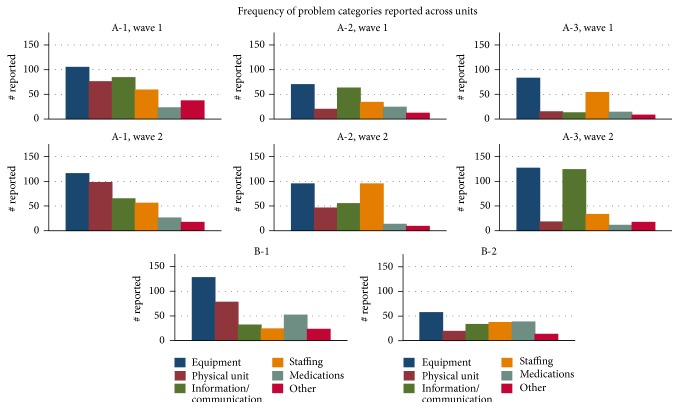
Frequencies for categories of small operational failures, disaggregated by unit and time.

The distribution of problem categories across the 5 units is displayed in Figure 3. In every clinical unit, operational failures related to equipment were the most commonly reported category. Comparing results from the first and second wave of data collection in Hospital A, the problem frequencies remain relatively consistent, with the exception of an increase in information/communication failures reported in the second wave on unit A-3 (*p* = 0.016).

It is important to note, however, that the 6 broad categories encompassed a large and diverse number of discrete small problems or operational failures. Across the 5 clinical units, the top 3 single problems accounted for 13 to 22% of the total on any one unit and the top 10 problems accounted for 32 to 43% of the total. Following the top 10 reported failures, there is a long tail of low frequency problems on each unit.  Table 3 lists the 10 most common operational failures reported across the 5 clinical units.

**Figure 4:**
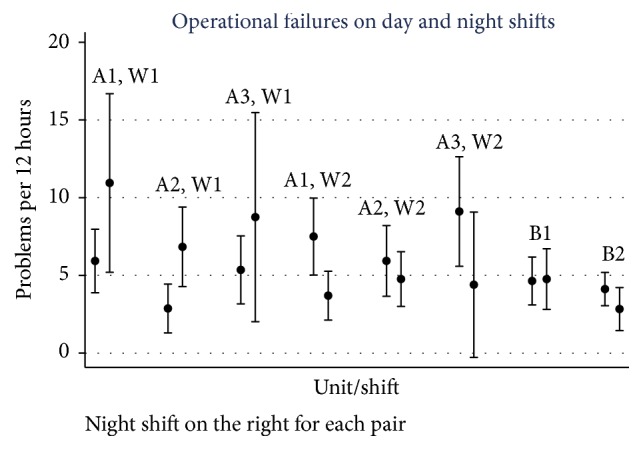
Rate of small operational failures by unit, disaggregated by day and night shifts, with estimate and 95% CI.

Comparing the rate of operational failures reported by day versus night shift discloses no statistically significant differences within units (Figure 4). In Hospital B, the absolute differences between day and night shifts within units are minimal. Larger differences in the number of failures reported in day versus night shifts are evident in Hospital A, although the direction of day to night differences is inconsistent across units and data collection periods (waves 1 and 2).

When we examined differences between nurses whose reporting frequency ranked at the top quartile with those in the bottom quartile, we found no systematic differences in the distribution of reporting categories. Nurses in the top quartile reported more failures across all categories, from one additional report/12 hours, on average, of a medication failure to about 3 additional reports/12 hours of equipment failure.

### 4.4. Unit Responses

The study timeline allowed only a short observation period for responses to the Pocket Card data at the 2 hospitals. Nonetheless, we did observe 2 units' responses to the Card data. One unit put up posters in the unit workroom displaying the most common operational failures so that nurses working both day and night shifts could vote for their highest priority problem. After choosing a problem, smaller subsets of the unit then met to select and implement strategies from the improvement toolbox. One unit focused on better managing their dirty utility room to reduce the time spent serially rearranging soiled equipment and supplies. Another unit used spaghetti diagrams to illustrate the maneuvers necessary to transfer patients from beds to gurneys in the cluttered patient rooms. They then suggested modifications to the standard layout of furniture in the rooms.

Upper-level nurse managers reviewed the operational failure data at each hospital but were not directly involved in formulating the improvement projects. Debriefing interviews with them at study conclusion did, however, suggest that they found the frontline perspective meaningful and a potential guide for action.

## 5. Discussion

The results of this exploratory study suggest that small operational failures are common on medical-surgical clinical units and it is feasible for unit staff to record these failures in real time. The 57.3% response rate suggests that the sample represents the whole staff; all categories of nursing staff participated in the identification of failures, with RNs being well-represented. Equipment problems are the most commonly reported failure, but many different types of failures are recognized on any single unit, so that even the top ten problems account for only about a third of the total number. On the units studied, small operational failures seem to be approximately as common during night shifts as day shifts. The categories provided on the Pocket Card seemed to be adequate for nurses to document in real time the type and frequency of operational failures. In fact, to ensure that all operational failures were recorded, the all-inclusive “other” category was also included on the Pocket Card. In all cases but one (unit A-1), the “other” category was the least used category for documenting the type and frequency of operational failures.

Compared with previous work using direct observation by an outside observer [12], nurse reporting yields about half the directly observed rate of 8.4 operational failures per 8-hour shift. Four potential reasons for underreporting in this study include the burden of recording problems during the flow of work, failure to recognize small operational failures because of desensitization to common problems, reinforcement for workarounds as solutions, and reluctance to report certain types of failures that may reflect badly on the unit or individual staff. Small problem data collected simultaneously by staff and third-party observers would help to distinguish these possibilities. Future research exploring self-detection versus observed detection of operational failures is necessary to expand our understanding of the variation of the two methods, including the identification of barriers to reporting operational failures.

These data are subject to other important limitations. First, our sample was limited to 5 clinical units in 2 hospitals; a much wider cross section of medical-surgical units will be necessary to understand the generalizability of our results. Second, limited participation rates among unit nursing staff raise the possibility of selection bias among nursing staff that chose to participate; participation may have been stifled by the perceived ability to extrapolate subject's identity from unit/shift/date information. Whether that bias may have acted to increase or decrease reporting is unknown. Third, as already noted, problem reporting depends on staff initiative, culture of accountability, and awareness to do so [10] and it may have underestimated the true occurrence of small problems. Healthcare professionals may not see quality improvement as part of their work and the structure and processes may not be in place to encourage such activity. The results may therefore be more valid as a description of the type of operational failures on a given unit rather than as an estimate of the true rate of operational failures. Fourth, staff on a given clinical unit form a social network, or microsystem, that influences the perceptions of operational failures, so that staff may be predisposed to noticing or reporting certain types of failures more frequently. Finally, but most importantly, the operational failure data are important only to the extent that they can be shown to drive meaningful quality improvement. Identifying operational failures is only the first step in a sequence that leads to effective interventions for improvement and redesign.

There is a strong theoretical basis for believing that data on operational failures are indeed important. Deming emphasized the crucial role of frontline workers in bringing process defects to the attention of management [17]. Later developments in quality improvement, such as Lean [11] and High Reliability Organizations [18], have continued to put frontline workers at the center of performance improvement. Emerging evidence provides support that engaging frontline nursing staff in identifying problems can lead to improved care quality. For instance, the Transforming Care at the Bedside (TCAB) initiative was designed to provide leadership support and tools for frontline nursing staff to develop, test, and implement care improvements [15, 19]. Among other outcomes, TCAB led to important reductions in patient fall injuries and codes [20].

Site reports on the type and frequency of operational failures were provided to each of the site clinical managers. In reviewing the report, clinical managers indicated that the Pocket Cards used in this study provided a useful, low-tech, dimension to assessing the fluidity of operations on the clinic floor that could not be captured by any other means. Future research could extend the detection of operational failures to an action-focused engagement of nursing management in initiating improvement to decrease operational failures.

Whether it makes sense to focus on small operational failures and the resulting workarounds, as opposed to more severe near misses or critical incidents, remains to be seen. The pervasiveness of workarounds suggests that they are an important feature of nursing work and thus shape the normative responsive to process defects [21]. But the large number and variety of different problems encountered in our study raises the risk of improvement fatigue. That is, we did not observe a classic Pareto distribution where a small set of failures makes up the majority; instead, the distribution displays a long tail of operational failures so that even the top 10 failure categories account for less than half the failures.

Continued research in the field of frontline engagement in the detection of operational failures is warranted. The results of this study provide preliminary evidence that frontline nurses can successfully engage in the detection first-order operational failures in real time. Furthermore, the results of this study suggest that the STAR Pocket Card can be a successful, low-tech, instrument in documenting and categorizing real-time operational failures that occur in healthcare.

Future research should expand upon this study with the addition of multiple hospitals and clinical units to produce greater generalizability of operational failure data. Future work should also investigate the role of contextual variables (i.e., Culture of Patient Safety, Team Vitality, and Job Satisfaction) and how they affect frontline engagement. As indicated previously in this paper, the detection and reporting of operational failures are a sensitive issue and nurses may perceive the reporting of large number of operational failures negatively to themselves or their clinical unit. By studying the contextual variables of the microsystem and system, a clearer picture can be painted on the role and impact these system variables have in the detection of operational failures. Future research should also include interventions to address the major operational failures reported in this study. The use of champions, coaches, or practice facilitators as part of an intervention warrants investigation at the microsystem level based on successes previously reported in improving quality of care in the primary care setting [22] and a 3-fold increase in the adoption of evidence-based guidelines [23].

## 6. Conclusion

The results of this pilot study provide preliminary evidence that frontline nurses are capable of engaging in the collection of real time operational failures that occur in the patient care process. All the categories that were listed as part of this study have direct impact on and affect the timeliness of care provided to the patient. The enormity of the variety of failures encountered by nurses during a shift provides opportunities for future research in this field to streamline the care process. Finally, the index-sized STAR Pocket Card proved to be useful as a low-tech device for the identification and documentation of operational failures, in real time, by clinicians.

## Figures and Tables

**Table 1:** Study participation.

Unit	Unit size	Consented	Participation rate
A1	47	25	53.2%
A2	42	27	64.3%
A3	52	35	67.3%

B1	95	50	52.6%
B2	43	23	53.5%

Total	279	160	57.3%

**Table 2:** Staff shifts, hours worked, and problems reported.

	Unit	Shifts^#^	Hours^#^	Problems	Problems/12 hours (95% CI)
Wave 1	A1	48	548	371	8.0 (5.3–10.8)
	A2	49	580	211	5.3 (2.9–5.8)
	A3	31	332	172	5.8 (3.7–7.9)

Wave 2	A1	66	776	367	5.6 (4.1–7.1)
	A2	57	680	313	5.5 (3.9–7.1)
	A3	36	420	278	8.5 (5.3–11.6)

	B1	89	880	333	4.7 (3.4–5.9)
	B2	53	584	194	4.0 (3.0–4.9)^*∗*^

^*∗*^Significant difference compared to the other units (*p* < 0.05).

^#^Significant difference between Wave 1 and Wave 2 for total number of shifts and hours reported.

**Table 3:** Most commonly reported operational failures.

Operational failure	Category	Count
Not enough PCAs/staff	Staffing	44
Redundant documentation	Communication	36
Illegible written orders	Communication	24
No communication about new admissions	Communication	19
Not enough vital sign machines	Equipment/supplies	16
Not enough IV pumps	Equipment/supplies	14
Not enough linens	Equipment/supplies	13
Dirty utility room	Physical unit/layout	13
Medication dispensation machine broken	Equipment/supplies	10
Scales too heavy	Equipment/supplies	9
